# Squamous Carcinoma of the Neovagina after Male-to-Female Reconstruction Surgery: A Case Report and Review of the Literature

**DOI:** 10.1155/2019/4820396

**Published:** 2019-01-16

**Authors:** Ronja Fierz, Gian-Piero Ghisu, Daniel Fink

**Affiliations:** Department of Gynaecology, University Hospital Zurich, Zurich, Switzerland

## Abstract

Squamous cell carcinoma (SCC) of the neovagina after genital reconstruction surgery is a rare occurrence with only very few cases published up to the present. We report a case of a 43-year-old transgender woman who developed neovaginal SCC 23 years after vaginoplasty. The patient tested positive for high-risk human papillomavirus (HPV). At the time of diagnosis, radiological investigations revealed already existing lymph node and osseous metastases. The treatment consisted of various cycles of chemotherapy and radiation therapy. Unfortunately, the formation of additional metastases, including cerebral, pulmonary, and hepatic metastases, could not be prevented. After comparing the literature on the topic, we conclude that neovaginal carcinoma often appears years and decades after genital reconstruction surgery. We therefore recommend the continuation of regular clinical follow-up for transgender women after postoperative follow-up is completed. With this approach, potential lesions may be detected at an earlier stage and a better outcome may be achieved. Follow-up should include neovaginal examination and cytological smear testing. At a later age, we recommend additional regular prostate-specific antigen (PSA) testing and digital rectal examination (DRE). Moreover, transgender women are advised to take part in mammography screening starting at the age of 50, especially when additional risk factors are present.

## 1. Introduction

SCC of the neovagina is a rare finding [1]. However, as genital reconstruction surgery becomes more accessible and is more frequently performed, awareness of the disease is important [2]. Vaginoplasty by penile or in combination with scrotal skin inversion is the commonly used technique in male-to-female reconstruction surgery [3]. Further methods of neovaginal construction include transposition of peritoneal, intestinal, and full or split skin grafts. These methods are also used for vaginoplasty after resection of a natural vagina or in women with vaginal agenesis as a result of Mayer-Rokitansky-Küster-Hauser syndrome [3, 4].

In this report, we present a case of neovaginal SCC arising in an HIV-positive transgender woman, which has also been mentioned in a recent publication by Grosse et al. [5]. The tumour developed 23 years after vaginoplasty, presumably as a consequence of high-risk HPV infection of the penile skin. In the literature review, a comparison was made between the present case and the three cases of SCC in the neovagina of transgender women published to date [6–8]. This was collated with a case series of 16 neovaginal carcinoma in nontransgender women with primary vaginal malformations, as reviewed by Steiner et al. [4].

## 2. Case Report

We report the case of a 43-year-old HIV-positive transgender woman. The patient presented at her physician in December 2015 after discovering intermittent vaginal bleeding for 4 weeks. For the same period of time, she stated a foul-smelling vaginal discharge and occasional pain in the genital region that increased while walking.

The patient had undergone genital reconstruction surgery in Thailand at the age of 21 and had been on hormone therapy ever since. Unfortunately, the documents of the procedure were unavailable. However, it seems highly probable that the neovagina was formed using penile and scrotal skin since clinical examination indicated a full skin graft. In addition, combined penile and scrotal skin inversion is the most frequently performed vaginoplasty in transgender women, as mentioned above. The first 11 years after the creation of the neovagina remained uneventful. The following year, the patient was diagnosed with an HIV infection CDC-classification stadium B3. She then received antiretroviral therapy, which she interrupted twice but with negative viral load at time of presentation. Several months after the HIV diagnosis, the patient suffered from a cohabitational injury, which was revised subsequently.

With the history of vaginal bleeding, her physician referred the patient to the gynaecology department of the University Hospital Zurich. Vaginal and speculum examination revealed an ulcerative lesion, which was painful on palpation and started bleeding in contact with a swab. The patient denied recent cohabitations and vaginal trauma.

**Figure 1:**
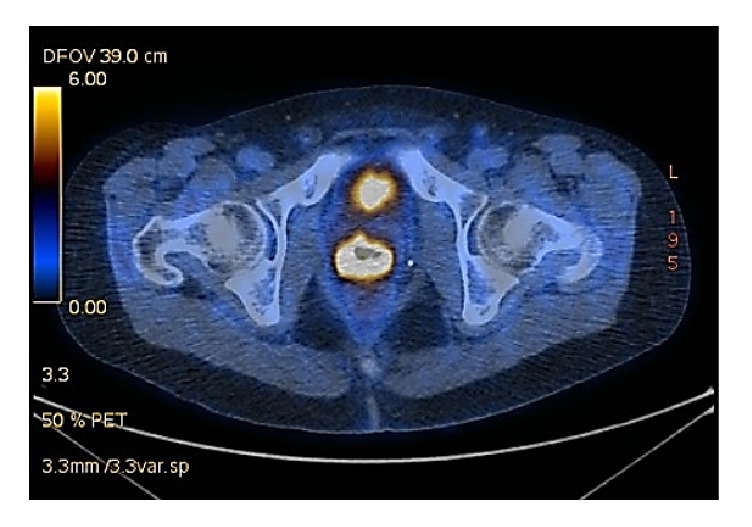


**Figure 2:**
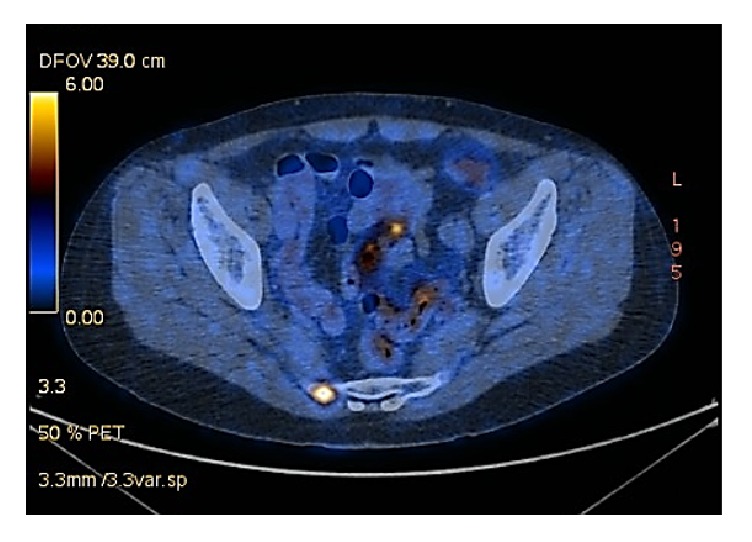


The swab of the lesion revealed a high-grade squamous intraepithelial lesion (HSIL). Subsequent MRI showed blood in the neovagina and could not exclude a neovaginal tumour. Nevertheless, no extravaginal masses or enlarged lymph nodes were detectable. PET-CT scanning was performed and revealed a distinctly progressive tumour at the apical anterior wall of the neovagina with a wall thickness of 1.3 cm (Figure 1). The mass was highly FDG-active and infiltrated the neovagina in a craniocaudal extension of at least 4.6 cm. The scan also showed a slightly enlarged left inguinal lymph node compared to the MRI scan performed before. Moreover, multiple FDG-active osseous metastases were visible, located at the 5^th^ right lateral rib, the 3^th^ lumbar vertebral body, and the right side of the sacral bone (Figure 2). Fine-needle biopsy confirmed the diagnosis of a high-grade squamous carcinoma.

In the given palliative situation, concomitant chemoradiotherapy was started with the aim of gaining locoregional tumour control. 6 cycles of cisplatin chemotherapy and percutaneous radiation therapy were conducted on the primary lesion, the pelvic lymph system, and the osseous metastases. This was followed by another 6 cycles of first-line chemotherapy. The patient already suffered from impaired general condition aside from the known comorbidities such as HIV infection. For this reason, the gynaecological tumour board discouraged a more aggressive chemotherapy usually applied in case of metastatic penile cancer. Six cycles of carboplatin/paclitaxel and concomitant bone protection (denosumab) were suggested instead. The PET-MRI scan after completion of chemotherapy showed a mixed response. It included regression of the primary lesion, a progressive hepatic metastasis, small lung, and several additional osseous metastases. Hence, selective internal radiation therapy was performed on the right hepatic lobe. A further approach was made, consisting of percutaneous radiotherapy on the osseous metastasis of the scapula. Unfortunately, continuous progression of the hepatic, osseous, and pulmonary metastases was visible in a PET-CT 2 weeks later, and the options of further treatment were profoundly discussed. Retrospectively performed PCR of the tissue sample, which was gained at the time of diagnostic biopsy, confirmed infection with high-risk HPV type 51. At this time, the patient was already in reduced general condition and did not qualify for another combination chemotherapy. Nevertheless, the patient explicitly wished to proceed with systemic therapy. A cycle of more tolerable topotecan monotherapy was therefore attempted, analogous to the treatment of SCC of the cervix. In addition, radiotherapy on the osseous metastases was continued. When the patient started to perceive dizziness shortly afterwards, a cystic cerebellar metastasis with partial compression of the 4^th^ ventricle was detected by MRI scan. To ease her symptoms, suboccipital craniotomy was performed with macroscopically complete resection of the lesion. Histopathology confirmed the diagnosis of a keratinizing SCC metastasis. At this point, our patient was, regrettably, in terminal stage. She was transferred to a Palliative Care Center, and we made efforts to relieve her from pain as much as possible. Shortly afterwards, during the process of this report, our patient passed away, 2 years after primary tumour diagnosis.

## 3. Review of the Literature

To our best knowledge, only 4 cases of neovaginal SCC in transgender women after genital reconstruction surgery have been published to date, including our patient's medical record [6–8]. The age at reconstruction was between 21 and 33 years, with a median of 27 years. In all the vaginoplasties, skin grafts were used to create the cavity of the neovagina. In two cases, the skin was taken from the penis and scrotum, which was presumably also the case in our patient's surgery. The 4^th^ patient initially also underwent vaginoplasty by penile and scrotal skin inversion. Besides, she had another surgery a year later with the aim of creating a deeper cavity of the neovagina. An abdominoperineal skin graft was used for this purpose. Unfortunately, it is unknown whether the first implant was excised completely or whether the second graft was added to the already existing neovaginal pouch. After 10 years, another two smaller revisions were performed. The age at diagnosis ranged from 42 to 78 years, with a median of 54 years. The latency period between the age at reconstruction and the age when diagnosed with cancer was between 18 and 45 years, with a median of 26.5 years. All patients reported vaginal discharge as first symptom. Two patients had an additional fistula, and two patients also stated vaginal bleeding. One patient claimed genital discomfort as another symptom. Physical examination revealed tumour masses, which were necrotic in one case and ulcerative in another. In two cases, the tumour was located at the posterior part of the neovagina. In a further case, it was situated at the apex and in our case the location of the tumour was the apical anterior wall of the neovagina (Table 1) [6–8].

The primary treatment of the neovaginal lesions was in two cases a combined chemoradiotherapy and in one case a tumour exenteration with subsequent chemotherapy. In the 4^th^ case, the neovagina was fully resected, with additional combined chemoradiotherapy. All four cancerous lesions were confirmed by histological examination to be due to SCC. Two patients were diagnosed with moderately differentiated skin cancer, one suffered from a well-differentiated carcinoma, and our case turned out to be a high-grade SCC. In two cases, the lymph nodes showed no enlargement. Our patient's lymph node biopsy was positive for cell aberration and the 4^th^ patient's lymph node state was not mentioned. The first two transgender women had a recurrence-free follow-up of 2 and 2.5 years, respectively. The patient with the well-differentiated carcinoma died after 2 months due to sepsis and multiorgan failure. And our patient had an aggravating clinical course over a time period of 2 years, including the development of multiple metastases, which she did not survive either. PCR of the neovaginal lesion biopsies was positive for HPV in three cases. Twice, a high-risk HPV type was detected (in one case type 16 and in our case type 51). In the 3^rd^ case, the HPV type was not specified in the paper. Unfortunately, PCR was not performed in the 4^th^ case. Nevertheless, immunohistochemistry for p16 turned out to be negative (Table 2) [6–8].

Based on the occurrence of HPV in three of the four published cases, it might be reasonably assumed that HPV plays a significant role in the development of SCC of the neovagina constructed by penile skin. This applies especially to high-risk types of HPV, as will be discussed in more detail.

In their case report “Carcinoma of the Neovagina: Case Report and Review of the Literature”, Steiner et al. analysed 16 cases of neovaginal carcinoma [4]. The patients were nontransgender women who were born with vaginal malformations. In their literature review, the women had mostly been operated at a very young age, whereby a functional neovagina was created. The median age at vaginoplasty was 18.1 years, whereas it was 27 years in our study. Moreover, the nontransgender women were also younger at the time of diagnosis, with a median of 36 years. In comparison, our patients were on average 54 years old when diagnosed with cancer. Thus, the latency period was shorter in their study, with a median of 17.4 years compared to the transgender women, who had an average latency period of 26.5 years. Ten of the 16 nontransgender patients reported vaginal bleeding and four also claimed discharge. In contrast, all of the transgender women named vaginal discharge as first symptom and two of the four also reported vaginal bleeding. The tumours of the nontransgender women were mainly located at the posterior wall and in two cases the lesions had developed at the apex of the vagina. This is consistent with our finding (two tumours at the posterior wall, one tumour at the apex, and one at the apical anterior wall) [4, 6–8].

In the study by Steiner et al., radiation was used in eight of the 16 nontransgender patients as the primary choice of treatment. A primary surgical approach was chosen in six cases, and in two cases there was no information regarding the primary treatment. In comparison, the transgender women were treated with combined chemoradiotherapy in half of the cases, whereas surgery was chosen as the primary treatment in the other half. Steiner et al. report that diverse methods of vaginoplasty were carried out. Skin transplants were used in seven cases, intestinal grafts in four cases, and peritoneal and dura tissue each in one case. Two women obtained vaginal construction without the use of a graft and once the transplant tissue was not described. Histopathology of the lesions identified 11 cases of SCC and five cases of adenocarcinoma. The tumour tissue in all four intestinal transplants turned out to be due to an adenocarcinoma. The 5^th^ case of adenocarcinoma, however, developed in a woman whose neovagina had been created using a skin graft only. Ten of the 11 SCC evolved from skin, peritoneal and dura grafts, as well as from neovaginal tissue formed by vaginal traction (without graft). The 11^th^ case appeared in the woman whose transplant tissue is unknown. However, the genital reconstruction surgeries of the four transgender women were performed using skin grafts only, and the biopsies of the lesions led to the diagnosis of SCC in all cases. In one of the nontransgender women in the study by Steiner et al., the lymph node state showed a pathological finding. In two cases, the examined cells showed no change in differentiation, and as for the other 13 women there was no information on the lymph node state available. In our study, abnormality of the lymph node tissue was detected in one transgender patient. Furthermore, there were two negative findings and one case without information on the topic. Five of the nontransgender women with vaginal agenesis were at time of publication of their cases in a disease-free follow-up state for several months. Three of the women died due to their condition and two women suffered from a recurrence within only a few months. Unfortunately, the clinical course of the other five nontransgender women is unknown. In comparison, two of the transgender women, including our patient, succumbed to their diseases. The other two, however, showed a disease-free interval of at least 2 years prior to the publication of their cases. Whereas HPV infection was present in three of the four transgender women, Steiner et al. did not detect any low-grade or high-grade HPV infection in the biopsy material of their patient. Unfortunately, the matter of HPV concerning the other 15 nontransgender women was not discussed in the paper [4, 6–8].

## 4. Discussion

As penile skin inversion is the most frequently applied technique in male-to-female reconstruction surgery, the original tissue of the newly formed vagina is exposed to a different environment [9]. The recently published study by Grosse et al., which examined the cytology of 20 neovaginal tissue samples in transgender and nontransgender women, discovered inflammation to be present in 40% of the cases [5]. The abnormal exposure of the neovaginal tissue therefore seems to be a risk factor for chronic irritation with subsequent inflammation and infection of the neovagina [2]. The regular use of a prosthesis for neovaginal dilatation and the higher neovaginal pH compared to nontransgender women were found to be further risk factors [2, 4]. Consequently, the graft tissue may be susceptible to sexually transmitted diseases (STDs) as well.

Hiroi et al. summarized 11 cases of neovaginal carcinogenesis in nontransgender women after vaginoplasty due to vaginal agenesis [10]. When interpreting their results, it occurred to them that all of the patients with skin transplants developed SCC of the neovagina. In contrast, every case of intestinal neovaginal graft mutated into an adenocarcinoma. These findings led to their conclusion that the tissue used in vaginoplasty has considerable influence on the direction of pathogenesis and the resulting malignant manifestation [10]. As explained above, the study of Steiner et al. showed similar results: All of the four women with intestinal grafts developed an adenocarcinoma, whereas SCC evolved in eleven of the twelve women, whose neovaginas were constructed by vaginal traction (no graft) or the use of skin, peritoneal and dura grafts. Nevertheless, one case of neovaginal skin transplant turned into an adenocarcinoma. However, the woman did present with a rectoneovaginal fistula at the time of diagnosis. Since the tumour was located at the posterior wall of the neovagina, it may be assumed that it evolved from the intestinal tissue of the colon rather than the neovaginal skin graft [4].

Studies have stated a high prevalence of sexually transmitted infections in transgender women [11]. These include HIV and HPV, which is the most frequent sexually transmitted infection [11–15]. Furthermore, it was reported that infection with a high-risk HPV type is not only essential to cervical carcinogenesis but also associated with anogenital and penile malignancies [16, 17]. Penile carcinomas are most commonly SCC emerging from penile intraepithelial neoplasia precursor lesions. In the formation of this tumour type, a high-risk HPV infection is assumed to be the cause of malignant transformation in about 40-50% of the cases [16, 17]. Anal carcinogenesis studies have shown that high-risk HPV, predominantly HPV type 16, is associated with about 90% of anal cancers [16]. These findings suggest that HPV infection plays an important role in male genital carcinogenesis as well. Since, histologically, neovaginal SCC of transgender women are SCC of the penile and scrotal skin grafts, the same applies to transgender women.

In contrast to nontransgender females, transgender women receive their neovaginal transplants almost exclusively from skin grafts [2, 3]. And since skin transplants seem likely to transition into SCC rather than adenocarcinoma, as previously discussed, HPV-associated neovaginal carcinogenesis might figure more prominently in transgender than nontransgender women. This is due to the fact that nontransgender women who were born with vaginal anomalies usually receive intestinal transplants, which are more likely to change into adenocarcinoma [4].

In addition, transgender women are at high risk of acquiring an HPV infection, since the prevalence of HPV is generally high in the male genital region (up to 76% [9]). Moreover, the majority of transgender women have a hetero- or bisexual orientation and are therefore likely to have sexual contacts with men [2, 9]. As unexpected pregnancy is not a risk, sexual protection may be used to a lesser extent than in nontransgender women. HPV and other STDs might therefore be more easily transmitted.

A recently published study, which analysed the prevalence of HPV infection in transsexual men and women, found HPV infection rate of 52.4% in the examined male-to-female transgender cohort [14]. Furthermore, and even more notably, 100% of the HPV-positive transgender women had been infected with at least one oncogenic HPV genotype. The study also stated that, in more than a 3^rd^ (35.7%) of the HPV-positive transgender individuals, vaccine-preventable HPV genotypes were detected. This demonstrates the importance of HPV vaccinations in the mentioned patient cohort [14].

However, prevention of neovaginal carcinogenesis in transgender women should be extended beyond the recommended vaccination program. Weyers et al. reported that only 4% of the examined individuals ever had a gynaecological exam after follow-up of vaginoplasty was completed [2]. As previously discussed, our observations suggest that neovaginal tumour masses are often located at the posterior wall or the apex of the neovagina. Detection by the patients themselves might therefore be a difficult matter, especially at an early stage of disease. At the same time, speculum and digital neovaginal exams were found to be well tolerated by transgender women [2]. Furthermore, regular gynaecological examination was considered by the majority (84%) to be an appreciated follow-up procedure after genital reconstruction surgery [2]. We therefore recommend the continuation of annual follow-up for transgender women after postoperative follow-up is completed, including speculum and digital neovaginal examination.

Although it is not generally recommended in medical checkup after genital reconstruction surgery, the new guidelines of the endocrinology society suggest routine cancer screening for transgender women [18]. Following the Swiss guidelines for nontransgender women, we primarily recommend cytologic smear testing every three years, starting at age 21 until patients reach the age of 70 [19].

As transgender women usually take feminizing hormones for years, a risk assessment for cardiovascular disease is recommended [2, 20]. Particularly the incidence of thromboembolic events may be a serious complication in patients with estrogen substitution therapy [18]. A further matter to be discussed in the individual patient's follow-up is mammography screening. Long-term use of estrogen may increase the risk of breast cancer development in transgender women, who are usually less affected than nontransgender women [2, 20]. We therefore suggest routine mammography screening every two years, starting at the age of 50 until 70, as it is clinical practice in University Hospital Zurich for nontransgender women [21]. Particularly individuals with a positive family history or further risk factors for breast cancer are advised to take part in regular screening [20, 21].

The prostate is generally left in place when vaginoplasty is performed [2]. Following the guidelines and clinical practice of University Hospital Zurich for cisgender men, we recommend PSA testing and DRE every four years (for PSA levels < 1ng/ml) in addition to the annual follow-up, when transgender women reach the age of 45 years [22].

## 5. Conclusion

In conclusion of our findings, we state that carcinogenesis in transgender women after genital reconstruction surgery may develop after a long latency period and with rapid progression. Adequate patient's instruction is therefore an important matter. We advise gynaecologists to encourage transgender women to undergo a continuous annual follow-up after postoperative follow-up is completed. Neovaginal carcinoma, as a rare condition, has been observed to be frequently located at the apex or the posterior wall of the neovagina. As a consequence, they may be difficult to discover by the patients themselves. Through regular follow-up, including neovaginal examination and cytological smear testing, the patients may be diagnosed at an earlier stage of carcinogenesis. Hereby the outcome may be positively affected. Considering the long-term hormonal substitution, which most of the transgender women make use of, potential thromboembolic events should be borne in mind. Starting at the age of 50, transgender women should take part in regular mammography screening, especially when additional risk factors are present. As the prostate is mostly left in place, regular PSA testing and DRE are valuable techniques in prostate screening and should be added to the annual follow-up when patients reach the age of 45.

## Figures and Tables

**Table 1:** Age at reconstruction and diagnosis; time between procedure, symptoms, and physical examination.

**Reference**	**Age at reconstruction (years)**	**Procedure**	**Age at diagnosis (years)**	**Years/between**	**Symptoms**	**Physical examination**
Y. Harder *et al.* [6]	24	Penile and scrotal skin inversion	42	18	Increasing vaginal discharge, fistula	Mass, posterior vagina

H. Fernandes *et al. *[7]	30, 31	Skin graft	53	21	Bloody, foul-smelling discharge	4 cm necrotic mass, apex of neovagina

J. Bollo *et al. *[8]	33	Penile and scrotal skin inversion	78	45	Genital discomfort, vaginal discharge	Bulky mass, posterior vagina

Present case	21	Presumably penile and scrotal skin inversion	43	22	Vaginal bleeding, foul-smelling discharge	4.6 cm ulcerative tumour, apical anterior vagina

**Table 2:** Primary treatment, histopathology of tumours and lymph nodes, results of treatment, and additional clinical data.

**Reference**	**Primary treatment**	**Pathology**	**Lymph nodes**	**Result/therapy**	**Additional clinical data**
Y. Harder *et al. *[6]	Total resection of neovagina followed by combined chemoradiotherapy	HPV-induced squamous cell carcinoma of the penis	No enlargement	Disease-free follow-up for 2.5 years	Previous history of chronic venereal warts (low-risk HPV infection)

H. Fernandes *et al*. [7]	Combined chemoradiotherapy	Moderately differentiated squamous cell carcinoma	No information	Disease-free follow-up for at least 2 years	No history of STIs including warts, negative p16 immunohistochemistry

J. Bollo *et al. *[8]	Tumour exenteration and chemotherapy	Well-differentiated squamous cell carcinoma	No enlargement	Death after 2 months	High-risk HPV (type 16) infection, death due to sepsis and multi-organ failure

Present case	Combined chemoradiotherapy	High grade squamous carcinoma	positive	Death 2 years after diagnosis	High-risk HPV (type 51) infection, HIV infection, multiple osseous metastases at time of diagnosis
